# FOLLOW-UP OF PATIENTS WITH POST COVID-19 CONDITION AFTER A MULTIDISCIPLINARY TEAM ASSESSMENT: A PILOT STUDY

**DOI:** 10.2340/jrm-cc.v7.24581

**Published:** 2024-09-23

**Authors:** Alexander WIGGE, Johanna PHILIPSON, Solveig HÄLLGREN, Helena FILIPSSON, Britt-Marie STÅLNACKE

**Affiliations:** 1 Department of Community Medicine and Rehabiliation, Rehabilitation Medicine, Umeå University, Umeå, Sweden; 2 Department of Clinical Sciences: Neurosciences, Umeå University, Umeå, Sweden

**Keywords:** post-COVID-19 condition, residual symptoms, rehabilitation

## Abstract

**Objective:**

To follow up patients with post-COVID-19 condition (PCC) 6 months after a multidisciplinary team assessment in specialist care regarding symptoms of pain, anxiety, depression, fatigue and cognition, level of activity, physical activity and sick leave.

**Methods:**

A prospective pilot study conducted in a clinical setting of patients (*n* = 22) with PCC referred from primary healthcare to a specialist clinic for a 2 day-multidisciplinary team assessment followed by a subsequent rehabilitation plan. Data were collected through questionnaires filled in prior to the team assessment and 6 months later.

**Results:**

Fifteen of the initial 22 patients participated in the follow-up. No statistically significant improvements were seen in any of the questionnaires after 6 months. However, 76.9% of the participants perceived the intervention as being helpful. This differed between the genders, where all the women 100% (*n* = 8) perceived it as being helpful, compared with 40% (*n* = 2) of the men (*p* = 0.012).

**Conclusions:**

Based on these findings, the benefit of a multidisciplinary team assessment of PCC is not fully convincing. However, since the participants themselves perceived the intervention as being helpful, the team assessment seems to be of some value. Further studies with larger populations would be of interest.

## LAY ABSTRACT

Most people who become ill with COVID-19 experience mild symptoms and recover within days or weeks. However, minor symptoms last from weeks to months and include fatigue, attention disorder, headache, dyspnea, anxiety, pain, anosmia, etc. This clinical state has been denoted as “post-COVID-19 condition” (PCC). It has been recommended that complex cases of PCC are to be assessed by a multidisciplinary team regarding treatment and rehabilitation. The efficiency of this recommendation has not yet been proven. This pilot study aimed to follow up patients with PCC six months after a multidisciplinary team assessment in specialist care regarding symptoms of pain, anxiety, depression, fatigue and cognition, level of activity, physical activity and sick leave through questionnaires. The results showed no statistically significant improvements in any of the questionnaires after six months. The majority of the participants perceived the team assessment as being helpful. Since the assessment seemed to be of some value, there is a need for further studies with larger populations.

At the end of 2019, a newly discovered coronavirus, Severe acute respiratory syndrome corona virus 2 (SARS-CoV-2), was found to be the causative agent of the disease COVID-19. Most people who became ill with COVID-19 experienced mild symptoms and recovered within days or weeks. However, a minority became seriously ill with a need for hospitalization (1). Global mortality has been estimated to be 1% and in Sweden 0.8% (2, 3).

Although a majority of those infected by SARS-CoV-2 returned to their normal state of health once the infection had been cleared, many patients continued to be symptomatic or developed new symptoms due to the infection. These sequelae can last for weeks to months, and include fatigue, attention disorder, headache, dyspnea, anxiety, pain, anosmia, ageusia etc. (4–6). This clinical state has been denoted as “post covid-19 condition” (PCC). According to WHO, PCC refers to long-term symptoms experienced after a confirmed or probable SARS CoV-2 infection. Individuals with PCC remain symptomatic or develop new symptoms within 3 months after falling ill. The symptoms persist for at least two months and cannot be explained by an alternative diagnosis (7).

Studies have shown that the prevalence of PCC varies. Some reports estimate that 3–10% have been suffering from symptoms of PCC for 12 weeks or more (8, 9). The severity of the acute disease has not been shown to affect the risk of acquiring PCC (10). However, it has been reported that the female sex, as well as increasing age and body mass index (BMI) are factors associated with increased risk of PCC (9).

The mechanisms of PCC are largely unknown. It has not been established whether parts of PCC are caused by the infection itself or if the condition is triggered by underlying chronic disorders. In addition, the effects of the pandemic on the individual have not been thoroughly investigated. The following are some of the hypotheses regarding the pathophysiology of PCC: harbouring of the virus in tissue reservoirs across the body; immune exhaustion with delayed viral clearance, hence causing chronic inflammation; and autoimmunity caused by cross-reactivity between SARS-CoV-2-specific antibodies and host proteins (11).

Since PCC can present a wide array of symptoms, the National Board of Health and Welfare in Sweden has recommended that complex cases of PCC are to be assessed by a multidisciplinary team regarding treatment and rehabilitation (12). The efficiency or efficacy of this recommendation has not yet been proven through clinical studies. Although a team assessment and rehabilitation should be based on a biopsychosocial approach, the length and intensity of a rehabilitation intervention for PCC has not been defined.

Therefore, this pilot study aimed to investigate the results of a 2-day multidisciplinary team assessment in a specialist care setting six months after the initial assessment with a focus on level of activity, sick leave, pain, anxiety, depression, fatigue and cognitive impairment. An additional aim was to study relationships between some of these variables.

## MATERIAL AND METHODS

### Design

A prospective cohort study.

### Setting

The pilot study was conducted in a clinical setting in specialist healthcare between May 17 and December 22, 2021, with pauses of a total of 7 weeks during the summer and autumn.

### Participants

Patients with PCS who were referred from primary healthcare centres in the County Council of Västerbotten to a multidisciplinary team at the neuro-head-neck centre (NHHC), Umeå University Hospital in Umeå, Sweden.

Inclusion criteria:

- Age at inclusion > 18 years, with a history of probable or confirmed SARS CoV-2 infection assessed at a primary healthcare centre.
- Remaining symptoms of pain or neurological deficits such as fatigue or cognitive difficulties, for more than 12 weeks after falling ill. Symptoms of at least a moderate degree and assessed to be caused, or clearly worsened, by the SARS CoV-2 infection.
- Unimodal treatment with no obvious effect, whereby specialized multidisciplinary assessment regarding rehabilitation was deemed as being necessary.

A total of 22 consecutive patients were included in the study.

### Procedure

Data were gathered in the form of questionnaires filled out at baseline assessment and at follow-up after six months. Participants were contacted by telephone and reminded about the questionnaires. Following this, 15 of the initial 22 participants filled out the questionnaires, while seven participants withdrew from the study. Data from questionnaires were complemented by data from medical records.

### Questionnaires

The *Occupational Gap Questionnaire (OGQ)* was used to measure participation in everyday activities (13). It consists of 30 items, each representing an activity. Every item is paired with two questions for the individual to answer. The questions are “Do you perform this activity?” and “Do you want to perform this activity?” Discrepancy between the two answers constitutes an occupational gap for that activity. Not want to do-gaps represent activities the individual does not want to perform but nevertheless does. Want to do-gaps are activities the individual wants to perform but nevertheless does not. These two kinds of gaps have been treated equally in the analysis, in accordance with previous literature (14). All items with incomplete or inexplicit answers in the OGQ were disregarded as missing data points.

The *Frändin Grimby activity scale* was used to assess level of physical activity (15). The questionnaire contains a 0–6 scale, with each number representing a degree of physical activity performed in everyday life. On this scale, 0 represents the lowest degree of physical activity and 6 the highest degree. Some participants marked two options on the scale. In these cases, the lower of the two values was chosen for analysis.

The *Hospital Anxiety and Depression Scale (HADS)* was used to assess anxiety and depression (16). It consists of 14 questions divided into two subscales: anxiety and depression. Each question has four alternatives with a score of 0–3 points. The total score range in both scales is 0–21. A score of 8–10 on one of the subscales suggests mild disorder, while a score > 10 indicates there is a clinically significant disorder (16).

The *Mental Fatigue Scale (MFS)* was used to assess symptoms of mental fatigue. It consists of 15 questions representing different symptoms of mental fatigue. Each question has seven alternative answers, where each alternative represents a certain number of points, ranging from no symptoms to severe symptoms. The cut-off score is 10.5 to distinguish between pathologic and non-pathologic mental fatigue. Scores exceeding 10 indicate that further investigation may be necessary. In this study, the MFS was only filled out at follow-up (17).

*A multiple-choice questionnaire (MCQ)* contained multiple-choice questions with 41 items. These questions focused on whether the individual experienced specific symptoms such as pain, fatigue, breathing difficulties or sleep disturbances. If they did, a quantifying alternative was to be chosen. There were also questions about work status and one question regarding whether they perceived the intervention with the 2-day multidisciplinary team assessment followed by a rehabilitation plan as being helpful. Nine items matching the aim of the study were chosen.

In the MCQ, answers in between options quantifying the given problem were counted as if the option representing the more serious problem had been chosen. All items with several answers, or items where the participant failed to choose a quantifying option, were disregarded as missing data points. The alternatives “minor problems” and “moderate problems” were grouped into the same category. For the drop-out analysis, the items were recoded into binary “yes” or “no” questions.

### Multidisciplinary assessment

Patients were assessed by a multidisciplinary team, which consisted of a physician, specialist in rehabilitation medicine, a neuropsychologist, a physiotherapist, an occupational therapist and a counsellor. All professionals had long experience of multidisciplinary team assessment and rehabilitation. Each assessment lasted from 1 to 2 h. The team assessment was based on the biopsychosocial approach, which considered physical, psychological and social aspects of the PCC condition. The physician’s assessment included a thorough medical examination with blood pressure and screening for orthostatic symptoms. The physiotherapist measured oxygen saturation at 1 min Sit-To-Stand Test and during 6-Min Walk Test. All the patients had normal levels of oxygen saturation during the tests. If there had been some questions and need for consultation a specific cardiologist was in service. Postural orthostatic tachycardia syndrome (POTS) was primarily investigated in primary care.

The individual assessment for each patient took place over the course of 2 consecutive days, during which the patients also filled out the questionnaires for the first time. At the end of day two, the team discussed their individual assessments to agree on a joint recommendation regarding rehabilitation. Afterward, the patients were invited to join the meeting where they were informed about the common assessment and recommendations made by the team. A rehabilitation plan was formed that included the team assessment of the patient´s condition and suggestions and recommendations for further rehabilitation. The individual rehabilitation plan was sent to the patient´s general practitioner to monitor.

Rehabilitation was then handled by the participants along with their respective primary healthcare centre for the following six months.

### Rehabilitation plan

The rehabilitation recommendations were based on individual needs. Some of the recurring recommendations were:

- Pulse-raising physical activity, where effort was to be limited by the degree of symptoms. When a certain level of effort had been feasible for a certain amount of time, effort could be increased slightly.
- Usage of cognitive aids for planning, such as calendars and lists.
- Regular meetings with a counsellor or psychologist.
- With the help of an occupational therapist, find a balance between activities that were strenuous for the participant and recovery.
- Gradual increase of workload.
- Regular mini-breaks at work to avoid triggering severe fatigue.
- Avoid exposure to excessive sensory stimuli in order to conserve energy.
- Pharmaceutical treatment of insomnia, pain and depressive symptoms.

### Data analysis

SPSS version 28.0.1.1 was used to process statistics. Non-parametric statistics were used for all the questionnaires. Descriptive statistics were used to describe the population. The Wilcoxon signed ranks test was used to compare results before assessment and at follow-up. Differences between the participants and the non-participants were analyzed using the Wilcoxon–Mann–Whitney test and the Chi-square test. Tests of correlation between variables were made with the Spearman correlation test. The *p*-value threshold was 0.05 throughout the study.

## RESULTS

### Drop-out analysis

At the time of multidisciplinary assessment, 22 patients participated in the study. By the time of the follow-up, seven had withdrawn from the study. There were no significant differences between participants and those who withdrew regarding age, gender, total HADS score and items from the MCQ.

### Demographics

Demographics are presented in Table I. Of those who provided information about hospitalization during infection, hospitalization occurred in 33.3% (*n* = 5). Three of the hospitalized patients provided information about BMI, all exceeding 25.

**Table I:** Demographics of participants

Variable	*n* = 15*	%	Median	IQR
Sex				
Men	6	40.0	-	-
Women	9	60.0	-	-
Age	-	-	45	10.0
Body mass index				
18.5–25	5	33.3	-	-
25–30	3	20.0	-	-
30–35	1	6.7	-	-
> 40	1	6.7	-	-
Missing data	5	33.3	-	-
Education				
Primary school	0	0	-	-
Secondary school	7	46.7	-	-
University	8	53.3	-	-
Time between infection and assessment				
Days**	-	-	313	222.5
Hospitalized during infection				
Yes	5	33.3	-	-
No	7	46.7	-	-
Missing data	3	20.0	-	-

* *n* = 15 was the total number of study participants.

** Calculating the median number of days between infection and assessment, data from 12/15 participants were used.

Previous cardiovascular disease (i.e. hypertonia, angina pectoris, atrial fibrillation, and pulmonary valve stenosis) was present in 33.3% (*n* = 5) of the participants. Asthma occurred in 20% (*n* = 3). A total of 26.7% (*n* = 4) had previously been diagnosed with mental health disorders including clinical burnout, depression and anxiety disorders. A total of 6.7% (*n* = 1) suffered from chronic pain.

### Outcome at 6-month follow-up

At the 6-month follow-up, there were no statistically significant changes regarding the total amount of occupational gaps in the OGQ (see Table II). When the results for men and women were examined separately, women reported more gaps at baseline assessment and follow-up than men. *Travelling* was the item with the highest frequency of gaps for all groups. Another top five item with respect to frequency was *helping and supporting others.* In relation to other items, this item increased in frequency at follow-up for every group (see Table III).

**Table II:** Comparison of data at baseline and follow-up

Instrument	All participants				*P*	Women				*P*	Men				*P*
	Baseline, *n* = 13–14		Follow-up, *n* = 13–14			Baseline, *n* = 8		Follow-up, *n* = 7–8			Baseline, *n* = 5–6		Follow-up, *n* = 6		
		
	Median	IQR	Median	IQR		Median	IQR	Median	IQR		Median	IQR	Median	IQR	
HADS-tot	9.0	14.5	10	16.75	0.593	9.0	8.25	8.5	7.75	0.733	14.0	21.5	17.5	19	0.144
HADS-A	6.0	9.0	5.0	10.5	0.504	5.5	4.75	3.5	5.5	0.785	8.0	13	10	11.75	0.279
HADS-D	6.0	6.5	5.0	5.0	0.876	5.5	5.5	5.0	3.25	0.336	6.0	8.5	7.0	6.5	0.129
Frändin Grimby	2.0	2.0	3.0	2.0	0.257	2.0	1.25	2.0	2.0	0.102	3.0	3.0	3.0	2.25	0.317
GAPS-tot	10.5	8.25	9.5	11.75	0.400	13.0	8.75	11.0	7.0	0.610	6.5	7.0	1.5	12.75	0.528
MFS			18.25	7.75				19.0	8.88				16.75	10.37	

HADS-tot – Total score on the Hospital Anxiety and Depression scale. HADS-A – Anxiety score on the Hospital Anxiety and Depression scale. HADS-D – Depression score on the Hospital Anxiety and Depression scale. Frändin Grimby – Score of physical activity on a 0–6 scale. GAPS-tot – Total number of occupational gaps from the Occupational Gap Questionnaire. MFS – Total score of points on the Mental Fatigue Scale.

**Table III:** Frequencies of occupational gaps for each individual item of the OGQ

Activity	Number of reported gaps				Number of reported gaps among women				Number of reported gaps among men			
	Baseline *n* = 11–14		Follow-up *n* = 11–14		Baseline *n* = 6–8		Follow-up *n* = 7–8		Baseline *n* = 4–6		Follow-up *n* = 4–6	
	*n*	%	*n*	%	*n*	%	*n*	%	*n*	%	*n*	%
Shopping for groceries	9	75	6	43	5	71	4	50	4	80	2	33
Cooking	4	33	5	38	3	43	4	50	1	20	1	20
Doing the laundry	4	30	5	36	2	29	3	38	2	33	2	33
Cleaning	5	36	6	50	4	50	5	71	1	17	1	20
Doing light maintenance	6	43	6	43	5	63	3	38	1	17	3	50
Doing extensive maintenance	7	54	6	46	6	75	5	71	1	20	1	17
Managing personal finances	1	7	1	7	0	0	1	13	1	17	0	0
Transporting oneself	6	43	4	29	4	50	3	38	2	33	1	17
Shopping	4	29	4	29	3	38	2	25	1	17	2	33
Participating/taking interest in sports/physical activity	8	62	7	50	6	86	6	75	2	33	1	17
Participating in outdoor activities	6	43	6	43	4	50	4	50	2	33	2	33
Having/practising hobbies	5	38	5	36	4	57	5	63	1	17	0	0
Participating in cultural activities	6	46	5	38	5	63	4	50	1	20	1	20
Watching TV/listening to the radio	2	14	0	0	2	25	0	0	0	0	0	0
Reading newspapers	2	14	2	15	2	25	1	14	0	0	1	17
Reading books/magazines	6	43	5	38	5	63	4	50	1	17	1	20
Writing emails, letters, books, poems	4	31	3	23	4	57	1	13	0	0	2	40
Playing games	5	42	5	38	4	57	4	50	1	20	1	20
Using computer and smartphone	0	0	1	8	0	0	1	13	0	0	0	0
Spending time with partner/children	0	0	0	0	0	0	0	0	0	0	0	0
Keeping contact/spending time with relatives, friends, neighbors	2	18	1	9	2	33	1	14	0	0	0	0
Helping and supporting others	9	64	8	62	6	75	6	75	3	50	2	40
Participating in local clubs’ activities	6	43	4	33	4	50	3	38	2	33	1	25
Practising religion	1	8	0	0	1	13	0	0	0	0	0	0
Visiting restaurants, cafés, pubs, dance clubs	8	66	6	50	5	71	4	57	3	60	2	40
Travelling	11	79	9	69	7	88	6	75	4	67	3	60
Working	5	36	6	46	4	50	4	50	1	17	2	40
Studying	4	29	4	33	2	25	3	38	2	33	1	25
Taking care of and raising children	1	8	0	0	1	14	0	0	0	0	0	0
Volunteering	6	43	5	42	4	50	4	50	2	33	1	25

OGQ: Occupational Gap Questionnaire.

There were no statistically significant changes in physical activity at baseline assessment compared with follow-up on the Frändin Grimby scale (see Table II).

Results from the HADS showed no significant changes either between baseline and follow-up for the whole population or between men and women. At baseline, 23.1% (*n* = 3) of the participants who answered (*n* = 13–14) had a score resembling a clinically significant disorder of anxiety and depression, respectively. At follow-up, these proportions were 28.6% (*n* = 4) for anxiety and 14.3% (*n* = 2) for depression. The HADS scores were generally higher among men than women, as seen in Table II.

The median value of fatigue on the MFS at follow-up was 18.25 (IQR = 7.75), which is above the cut-off value of 10.5 points. The median scores for men and women separately were also above 10.5 points, as shown in Table II.

An analysis of the separate items chosen from the MCQ, as shown in Table IV, indicated no significant changes between baseline and follow-up for any of the items. Concerning the item fatigue, 100% of those who answered, both at baseline and follow-up, reported that they suffered problems of fatigue.

**Table IV:** Comparison of data depicting job status and self-experienced issues from MCQ

Item	All participants				*P*	Women				*P*	Men				*P*
	Baseline, *n* = 11–12		Follow-up *n* = 13–15			Baseline, *n* = 8		Follow-up n = 8–9			Baseline *n* = 3–4		Follow-up *n* = 5–6		
	*n*	%	*n*	%		*n*	%	*n*	%		*n*	%	*n*	%	
Problems with daily life activities	12	100	12	80	0.679	8	100	8	88.9	1.000	4	100	4	66.7	0.655
Small-moderate	5	41.7	8	53.3		3	37.5	5	55.6		2	50	3	50	
Big	7	58.3	4	26.7		5	62.5	3	33.3		2	50	1	16.7	
Problems with pain and sensation	10	90.9	11	73.3	0.892	8	100	6	66.7	0.458	2	66.7	5	83.3	0.317
Small-moderate	4	36.4	7	46.7		3	37.5	3	33.3		1	33.3	4	66.7	
Big	6	54.5	4	26.7		5	62.5	3	33.3		1	33.3	1	16.7	
Problems with cognition	12	100	14	93.3	0.414	8	100	8	88.9	0.564	4	100	6	100	0.564
Small-moderate	3	25	8	53.3		2	25	5	55.6		1	25	3	50	
Big	9	75	6	40		6	75	3	33.3		3	75	3	50	
Problems with fatigue	12	100	15	100	0.564	8	100	9	100	1.000	4	100	6	100	0.317
Small-moderate	5	41.7	5	33.3		3	37.5	3	33.3		2	50	2	33.3	
Big	7	58.3	10	66.7		5	62.5	6	66.7		2	50	4	66.7	
Perceived helpfulness of intervention			10	76.9				8	100				2	40	
Small-moderate			9	69.2				8	100				1	20	
Big			1	7.7				0	0				1	20	
Employed	11	91.7	11	78.6	0.317	8	100	7	87.5	0.564	3	75	4	66.7	0.317
Full-time (100%)	7	58.3	6	42.9		4	50	3	37.5		3	75	3	50	
Part-time (25–75%)	4	33.3	5	35.7		4	50	4	50		0	0	1	16.7	
Sick leave	10	83.3	9	60	0.187	7	87.5	7	77.8	0.480	3	75	2	33.3	0.180
Full-time (100%)	6	50	5	33.3		5	62.5	4	44.4		1	25	1	16.7	
Part-time (25–75%)	4	33.3	4	26.7		2	25	3	33.3		2	50	1	16.7	
Early retirement	2	16.7	2	13.3	1.000	1	12.5	1	11.1	1.000	1	25	1	16.7	1.000
Full-time (100%)	1	8.3	1	6.7		0	0	0	0		1	25	1	16.7	
Part-time (25–75%)	1	8.3	1	6.7		1	12.5	1	11.1		0	0	0	0	
Sickness compensation	0	0	3	21.4	0.157	0	0	1	11.1	0.317	0	0	2	40	0.317

MCQ: multiple-choice questionnaire.

All of the patients who had reported on the item regarding cognition, had experienced such issues at baseline, while 93.3% had cognition issues at follow-up

Regarding the question whether patients perceived the 2-day team assessment followed by a rehabilitation plan as being helpful, 100% of the women who answered the question reported it was helpful, while the proportion of men who found it helpful was 40% (*p* = 0.012).

A majority of the participants were on some degree of sick leave, but there was no statistically significant change between baseline assessment and follow-up.

### Correlations

A statistically significant positive correlation was seen for the patients at follow-up (*n* = 13–14) between fatigue scores on MFS and depression scores on HADS (*r* = 0.716, *p* = 0.004). No significant correlation was found between the scores on MFS and the anxiety scores on HADS.

There was a statistically significant negative correlation between the scores for physical activity and total number of occupational gaps for the patients (*n* = 11–14) both at baseline (*r* = -0.761, *p* = 0.004) and follow-up (*r* = -0.733, *p* = 0.004). A significant negative correlation was found between age and total number of occupational gaps at baseline (*r* = -0.556, *p* = 0.039), but not at follow-up.

## DISCUSSION

The aim of the study was to follow up patients with PCC six months after a 2-day multidisciplinary team assessment in specialist care regarding level of activity, sick leave, pain, anxiety, depression, fatigue and cognitive issues. No statistically significant changes were seen on any of the measurement instruments when comparing baseline assessment and follow-up. Fatigue and cognitive impairment occurred widely both at assessment and at follow-up. Anxiety scores on the HADS resembled a mild anxiety disorder among men both at baseline and at follow-up. However, a majority of the participants perceived the intervention with the team assessment followed by a rehabilitation plan as being helpful.

The severity of the acute state of infection varied among the participants, where some had needed hospitalized care but others had not. This is in line with previous studies, showing that severity of disease does not seem to affect the risk of acquiring PCC (10, 18).

The prevalence of depression among participants in our study was almost twice as high compared with the average life-time risk of depression shown in a cross-national study (19). Another study of patients with COVID-19 has shown that previous depression, anxiety, and usage of antidepressants were risk factors for acquiring persistent fatigue 10 weeks after the acute state of infection (20). It is uncertain whether the prevalence in our study was high because of a non-representative population, or whether it corresponds with the claim of previous mental health disorders constituting risk factors. However, similar results were found in a study by Poyraz et al. where 284 patients were assessed 50 days after acute SARS-CoV-2 infection (21). The mean scores for anxiety and depression, as well as the proportions of clinically significant anxiety and depression disorders, were quite close to the proportions shown in our study.

Observed HADS scores of depression, and especially anxiety, were generally higher among men than women. This finding is in contrast with most previous studies about the epidemiology of these conditions, where women have been shown to run a higher risk of developing depressive disorders as well as anxiety disorders (22). The anxiety scores among men in our study were similar to those reported in a study on chronic pain (23), indicating that the levels of anxiety might be comparable in PCC and chronic pain at least in men.

There was a positive relationship between the scores for depression and fatigue in our study. A previous study has suggested a comorbidity of depression and fatigue, with partly overlapping symptoms (24). The correlation seen in our study might be applicable in a clinical setting, by potentially improving problems of fatigue through the treatment of depression. It is also worth noting that the median value on MFS was higher than the cut-off 10.5, which indicates pathologic mental fatigue.

The high levels of fatigue and cognitive impairment in our study, as shown by the scores on MFS and the MCQ, were about three to four times higher than the results in a systematic review by Ceban et al. in patients 12 weeks after acute COVID-19 infection (25). However, these differences might be due to the fact that one of the inclusion criteria in our study was neurological symptoms, which could have affected the prevalence of both fatigue and cognitive impairment in our sample.

Regarding the total number of occupational gaps on the OGQ, there were no significant changes between baseline assessment and follow-up. However, there was an interesting change of frequencies when looking at *helping and supporting others.* At follow-up, this item increased in frequency in relation to other items. At the same time, items such as *shopping for groceries* and *doing extensive maintenance* decreased in frequency in relation to other items at follow-up. These changes depicted a rise in the wish to help others, in relation to personal chores. This could indicate that the participants had an easier time carrying out personal chores at follow-up, and thereby an urge to spend more energy on helping others.

Physical activity, as measured by the Frändin Grimby scale, showed no statistically significant changes between baseline assessment and follow-up. In the literature, there are documented positive effects of physical activity for several disorders and conditions, for instance, mental health disorders and fatigue (26, 27). One could therefore speculate that increased physical activity might have led to more overall improvements in our population. However, the fatigue itself could be a reason why physical activity did not improve to begin with.

Despite the fact that no significant improvements were shown, a majority of the participants perceived the intervention with the 2 day-multidisciplinary team assessment followed by a subsequent rehabilitation plan as being helpful. Potential reasons for this seemingly contradictory result could be the feeling/experience of being looked out for and taken seriously. Even though our results do not indicate any significant improvements after the assessment, there might be other aspects of the rehabilitation interventions that provide helpful coping strategies, thus conveying the impression that the intervention was helpful.

There was a statistically significant difference between men and women, where a larger proportion of women perceived the intervention as being helpful. This kind of disparity between genders has been shown in multimodal rehabilitation of chronic pain, with larger improvements in women compared with men (28). In a previous quality study by Stenberg et al. (29) of neck and back patients it was shown that gender seemed to affect expectations and experiences of healthcare generally. In their study the men did not doubt that they were entitled to be helped, and took help from healthcare for granted while more women sought help for problems they had experienced for a long time but could no longer control. They expressed the attitude that others might have had a greater need of healthcare and doubted the value of their pain in the eyes of others.

We could only speculate whether this could be a factor that has influenced the results of our study.

However, there is an increasing knowledge about the natural course of post-COVID-19 symptoms. In a recent study from Japan of the 1391 adult patients diagnosed with COVID-19 it was revealed that the prevalence of post-COVID-19 symptoms after three months were 47.6% and after 1 year 31% (30). A minor proportion (12.6%) experiences symptoms lasting for over three months that interfered with daily life, however, based on these findings this study was conducted.

While assessing the quality of this study, several factors must be considered. Some strengths are that the multidisciplinary team consisted of experienced clinicians and that several of the questionnaires have been used in previous studies on similar groups of patients. Although a team assessment and rehabilitation should be based on a biopsychosocial approach, the optimal duration and intensity of a rehabilitation intervention for PCC has not been defined. In contrast to the short assessments that are commonly offered in ordinary healthcare the well-planned multidisciplinary assessment in this study was 2 days.

Some limitations to consider are the lack of power calculation, as well as the small size of the population following the exclusion of many of the referred patients. The small population in turn could be a reason for results that lack statistical significance. A final factor to be taken into account is that the compliance of the rehabilitation in the rehabilitation plan was unknown since this was dealt with by the patients themselves along with their general practitioner at the primary healthcare centres. However, since the referring general practitioners had experiences from patients with chronic pain and were used to the process of a 2-day assessment and rehabilitation plans it was possible to assume that the rehabilitation plans would be monitored. There was no control nor follow-up questions regarding compliance.

This study showed no statistically significant improvements in PCC following multidisciplinary team assessment and a rehabilitation plan. Nevertheless, a majority of the participants found the intervention helpful. However, more research on the subject is needed, preferably studies on larger populations, with control groups and compliance controls. In addition, qualitative studies may answer why women scored the assessment as helpful despite the absence of self-scored improvements.
